# Influence of Ethanol Parametrization on Diffusion Coefficients Using OPLS-AA Force Field

**DOI:** 10.3390/ijms24087316

**Published:** 2023-04-15

**Authors:** Bruno Zêzere, Tiago V. B. Fonseca, Inês Portugal, Mário M. Q. Simões, Carlos M. Silva, José R. B. Gomes

**Affiliations:** 1 CICECO—Aveiro Institute of Materials, Department of Chemistry, University of Aveiro, Campus Universitário de Santiago, 3810-193 Aveiro, Portugal; 2 LAQV-REQUIMTE, Department of Chemistry, University of Aveiro, Campus Universitário de Santiago, 3810-193 Aveiro, Portugal

**Keywords:** diffusion coefficient, liquid ethanol, molecular dynamics simulations, OPLS-AA

## Abstract

Molecular dynamics simulations employing the all-atom optimized potential for liquid simulations (OPLS-AA) force field were performed for determining self-diffusion coefficients ($D_{11}$) of ethanol and tracer diffusion coefficients ($D_{12}$) of solutes in ethanol at several temperature and pressure conditions. For simulations employing the original OPLS-AA diameter of ethanol’s oxygen atom ($\sigma_{\mathrm{OH}}$), calculated and experimental diffusivities of protic solutes differed by more than 25%. To correct this behavior, the $\sigma_{\mathrm{OH}}$ was reoptimized using the experimental $D_{12}$ of quercetin and of gallic acid in liquid ethanol as benchmarks. A substantial improvement of the calculated diffusivities was found by changing $\sigma_{\mathrm{OH}}$ from its original value (0.312 nm) to 0.306 nm, with average absolute relative deviations (AARD) of 3.71% and 4.59% for quercetin and gallic acid, respectively. The new $\sigma_{\mathrm{OH}}$ value was further tested by computing $D_{12}$ of ibuprofen and butan-1-ol in liquid ethanol with AARDs of 1.55% and 4.81%, respectively. A significant improvement was also obtained for the $D_{11}$ of ethanol with AARD = 3.51%. It was also demonstrated that in the case of diffusion coefficients of non-polar solutes in ethanol, the original $\sigma_{\mathrm{OH}}=0.312$ nm should be used for better agreement with experiment. If equilibrium properties such as enthalpy of vaporization and density are estimated, the original diameter should be once again adopted.

## 1. Introduction

Tracer binary diffusion coefficients ($D_{12}$) are essential when designing equipment or optimizing production, both for conventional and newly developed rate-controlled processes [1,2]. Even though large databases of diffusion coefficients have been published [3], a lack of data is still verified, especially for bioactive polar solutes in polar dense solvents such as ethanol.

The predominant method for experimental determination of $D_{12}$ values is the Taylor dispersion or Chromatographic Peak-Broadening method [4,5,6,7,8,9,10], which is time-consuming and requires specialized equipment and expensive solute standards. Alternatively, one may recur to phenomenological models such as the widely known Wilke–Chang equation [11,12]. However, for polar-solvent systems, the results achieved by such purely predictive equations are often moderate [3,13] since these models do not account for strong interactions between the molecules, for example hydrogen bonds. Data-correlative models, such as the 2-parameter equations of Dymond–Hildebrand–Batschinski (DHB) [14,15,16] and the Rice and Gray-based approach by Zêzere et al. [3], tend to yield better results, but their big disadvantage is the need of experimental data to determine the optimized parameters for each system.

An alternative approach for predicting $D_{12}$ considers Machine Learning (ML) algorithms as, for instance, the model proposed by Aniceto et al. [13] for polar-solvent systems. Although low deviations were achieved (average deviations of 5.07%), this approach has yet to withstand the test of time since most of these algorithms work as a “black box”, being hard to anticipate the magnitude of the deviations for a given system. Nevertheless, this is currently the best option for a “quick and dirty” estimation of $D_{12}$ of a polar-solvent system and should not be disregarded. ML models have also been developed for the $D_{12}$ prediction of non-polar systems [13] and of different solutes in supercritical CO_2_ [17].

Other approaches include Artificial Neural Networks (ANN) and Molecular Dynamics (MD) simulations. ANN have been employed for the prediction of self-diffusion coefficients ($D_{11}$) in pure liquids [18] and in binary fluid mixtures [19], whereas MD simulations have been successfully used, e.g., for the estimation of $D_{12}$, both in liquids [20] and in supercritical fluids [21], and of $D_{11}$ in liquids [22,23].

MD has been used to simulate the dynamics of molecular systems within a given period of time, by numerically integrating Newton’s equations of motion and upon consideration of quantum or classical mechanics to calculate the forces between the particles in the molecular systems under study, leading to the so-called ab initio MD (AIMD) or classical MD approaches, respectively, with the former being much more time consuming than the latter [24]. Classical MD simulations rely on sets of empirical parameters, a.k.a. force fields, to calculate the intramolecular and intermolecular interactions between the constituting particles in a specific system. Different degrees of empiricism originate different force fields, and different parametrizations define the limits of their application. There has been a continuous effort to develop new, or improve existing, force fields, which can be (i) coarse-grained, such as MARTINI [25], where groups of several atoms are represented by a single bead; (ii) united-atom, such as United-Atom Transferable Potential for Phase Equilibria (TraPPE-UA) [26], where, for example, hydrogen containing moieties as CH, CH_2_, and CH_3_ groups are treated as single unified interaction centers with masses corresponding to the sum of the masses of C and H atoms in the CH_x_ group; and (iii) all-atom, such as the General AMBER Force Field (GAFF) [27] and the All-Atom Optimized Potential for Liquid Simulations (OPLS-AA) [28], where all atoms are represented individually. The force fields are under continuous improvement, and new versions are published on a regular basis. For example, OPLS-AA has been the cornerstone of other force fields, e.g., L-OPLS-AA [29,30], OPLS-AA/M [31], and OPLS4 [32], which were introduced to overcome some limitations of the original set of parameters. The L-OPLS-AA enabled a more accurate reproduction of liquid properties of long alkanes, alcohols, esters, and glyceryl monooleate that was not possible using the OPLS-AA [29,30]; the OPLS-AA/M improved on previous iterations of the OPLS-AA force fields regarding its ability to reproduce both gas-phase conformer energies for longer peptides and aqueous phase experimental properties [31]; in OPLS4, the new parameters allowed for more accurately predicting protein–ligand binding affinities by addressing limitations in the representation of molecular ions, sulfides, and aryl sulfur and a general improvement in model hydration [32]. Consequently, the accuracy and the precision of the simulations tend to improve with time, which combined with the reducing costs of increasing computational power and efficient algorithms, make classical MD simulation an enticing approach for a $D_{12}$ estimation that, in some cases, may even compete with experimental studies.

The main focus of this work was to assess (and refine) the performance of OPLS-AA [28] for the calculation of transport properties, namely, $D_{12}$ of specific solutes in liquid ethanol and $D_{11}$ of ethanol. OPLS-AA was chosen due to its good description of liquid organic systems [33], the vast number of parameters available, compatibility, and easiness of implementation. Furthermore, some authors have shown that OPLS-AA can provide good results for $D_{12}$, for example Zêzere et al. [20], who estimated $D_{12}$ of ketones and aldehydes in pressurized liquid ethanol and by Vaz et al. [21], who estimated $D_{12}$ of ketones in SC-CO_2_. However, have regard for the computation of $D_{12}$ of protic solutes in ethanol and $D_{11}$ of ethanol [34], as these properties tend to be overestimated when using OPLS-AA, as will be presented further in this work. At this point, it is important to stress that the determination of accurate $D_{12}$ of protic solutes in liquid ethanol is of utmost importance to design and optimize industrial equipment and kinetic processes. Despite some correlations available in the literature [14,15,16], including some originating in our group [3], the fact is that there is no general theory to accurately estimate $D_{12}$ of polar systems without having experimental (or accurate computational) data. Therefore, new approaches for determining such values are expected to have an impact on the field.

Several modifications to OPLS-AA have been published to improve $D_{11}$ calculations of ethanol and other alcohols. For instance, Kulschewski and Pleiss [34] proposed the adjustment of the hydroxyl group partial charges to better describe $D_{11}$ of alcohols, but this parametrization still originated significant differences (e.g., for ethanol, the estimation of $D_{11}$ differs up to 22%, depending on the experimental value used for comparison). Another very recent work by Zhang et al. [35] focusing on C1 to C10 primary alcohols considered a combination of L-OPLS parameters for the hydrocarbon tail and OPLS-AA parameters for the hydroxyl group, as suggested by Zangi [33], with additional tiny adjustments (scale factors in the range of 1.00–1.03) of the partial charges. This approach, the so-called mixed-OPLS-AA model refinement of the OPLS-AA force field for liquid alcohols with scaled charges [34], decreased the deviations between experimental and estimated $D_{11}$ to values in the range of −8% for nonan-1-ol and 5% for ethanol [35]. For comparison, the corresponding deviations calculated with the original mixed-OPLS-AA model [33] (i.e., without the charge scaling) were 19% and 34%, respectively. Other force fields, such as OPLS4 [32], extensively tested by Baba et al. [22] for the prediction of $D_{11}$ over 152 diverse pure liquids at various temperatures, achieved maximum deviations roughly under 20% for ethanol. Petravic and Delhommelle [36] tested OPLS-UA (united atom version of OPLS-AA), which, similarly to OPLS-AA, overestimates $D_{11}$ of ethanol by roughly 25%, on average, due to density underestimation (around −4%). Enforcing the experimental density of ethanol decreased the $D_{11}$ value; the calculated value still slightly overestimates the experimental result, yet to a smaller extent [36]. Cardona et al. [37] tested both GAFF and TraPPE-UA for the estimation of $D_{11}$ for ethanol, reporting deviations of −2.08% and 7.47%, respectively. Finally, Schnabel et al. [38] proposed an ethanol rigid anisotropic united-atom model, based on Lennard-Jones and Coulombic interactions, which achieved average deviations of −6% for $D_{11}$ of liquid ethanol [39].

Not disregarding the previous proposed corrections and/or parameterizations, a different approach for refinement of OPLS-AA when computing either $D_{12}$ or $D_{11}$ is presented in this work. In particular, the influence of the diameter of ethanol’s oxygen atom ($\sigma_{\mathrm{OH}}$) on the diffusion coefficients of systems embodying hydrogen-bonding solvents is analyzed. This correction was triggered by Hirschfelder et al. [40], who claimed that the diameters of molecules tend to be smaller when computed from transport properties than when equilibrium properties are used. Here, we show that a reparameterization, i.e., smaller ethanol $\sigma_{\mathrm{OH}}$ value, can be successfully introduced in the case of self- and binary diffusion coefficients (transport properties) in opposition to equilibrium properties (namely, density and enthalpy of vaporization) for which the original parametrization affords better results. A database of experimental $D_{11}$ and $D_{12}$ values, together with the above-mentioned equilibrium data, was compiled in order to validate the MD simulations and assumptions.

## 2. Results and Discussion

### 2.1. D_12_ of Quercetin and Gallic Acid in Liquid Ethanol: Optimization of the Oxygen’s Radius

MD simulations of diffusion coefficients of polar solutes containing OH groups in liquid ethanol consistently generate $D_{12}$ with high deviations in relation to experimental data. For example, for quercetin in liquid ethanol at 1 bar modelled with the original OPLS-AA parameters, deviations of 26.61% and 31.78% were found at 303.15 K and 323.15 K, respectively. Similarly, for gallic acid at the same conditions, the deviations were 29.13% and 35.25% at 303.15 K and 333.15 K, respectively. Noteworthy, in both cases, the deviations increase with temperature, as previously reported for $D_{12}$ calculations of ketones and aldehydes in liquid ethanol [20]. The deviations are also consistent with those from previous MD studies focusing on diffusivities of alcohols, as summarized in the Introduction section.

**Figure 1:**
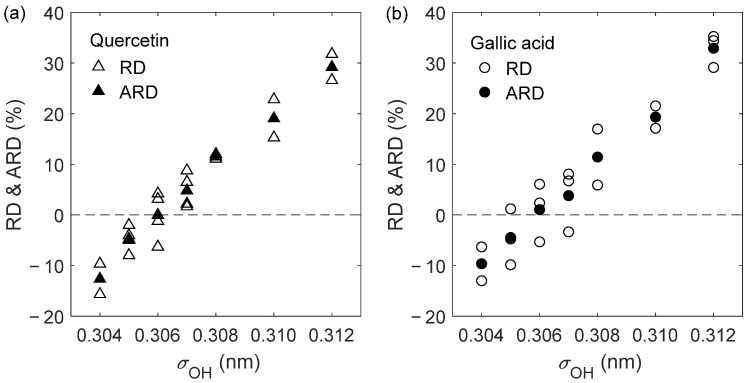
Relative deviations (RD) of $D_{12}$ of (**a**) quercetin (triangles) and of (**b**) gallic acid (circles) in liquid ethanol versus ethanol’s oxygen diameter ($\sigma_{\mathrm{OH}}$). Empty symbols are individual RD values at different T and P conditions, while filled symbols are average relative deviations computed from the RD values obtained at each condition.

Hirschfelder et al. [40] stated that the diameters of molecules tend to be smaller when computed based on transport properties than when using equilibrium properties. Therefore, we decided to investigate the effects of changing the diameter of the oxygen atom from the ethanol hydroxyl group upon the $D_{12}$ values derived from classical MD simulations. In the OPLS-AA force field, the diameter of the aliphatic alcohol oxygen atom ($\sigma_{\mathrm{OH}}$) is 0.312 nm. Thus, we performed simulations of ethanolic solutions of quercetin and gallic acid at the conditions reported in Table 1, using $\sigma_{\mathrm{OH}}$ for ethanol in the range 0.304 to 0.312 nm and without changing the remaining simulation parameters (Figure 1 and Table S1). The best comparison of calculated and experimental $D_{12}$ of quercetin and gallic acid in ethanol (Table 1) was obtained when using $\sigma_{\mathrm{OH}}$ = 0.306 nm. Encouragingly, the optimum diameter found is close to the 0.307 nm utilized in the OPLS-UA force field [28]. In practice, this change implies that the molecules of ethanol become closer to each other than in the original parametrization; hence, density increases (as it will be shown further) and the free volume of the solvent decreases, lowering the $D_{12}$ values. In the case of quercetin, at the simulated conditions of 303.15 K and 1 bar, 303.15 K and 150 bar, 323.15 K and 1 bar, and 323.15 K and 150 bar, the newly found deviations ranged between −6.30% and 4.22% (individual results are given in Table 1), with global average relative deviations (ARD, defined in Section 3.1) and average absolute relative deviations (AARD, defined in Section 3.1) of −0.04% and 3.71%, respectively. This is a massive improvement over the results achieved with the initial value $\sigma_{\mathrm{OH}}$ = 0.312 nm. A similar improvement was confirmed for gallic acid with the new $\sigma_{\mathrm{OH}}$ value giving relative deviations (RD, defined in Section 3.1) between −5.31% and 6.08% and ARD and AARD values of 1.05% and 4.59%, respectively. Although the ARD value is close to zero, for both solutes, a strong dependence between RD and T is verified with the $D_{12}$ values being slightly underestimated at 303.15 K and overestimated at 323.15 K and 333.15 K, as can be seen in Table 1. 

**Figure 2:**
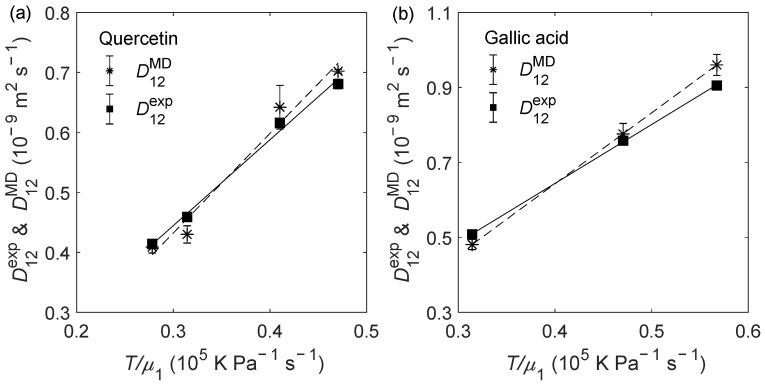
Experimental ($D_{12}^{\exp}$, ■) and computed ($D_{12}^{\mathrm{MD}}$, *) diffusion coefficients in liquid ethanol versus Stokes–Einstein abscissae ($T/\mu_{1}$): (**a**) quercetin, and (**b**) gallic acid. The viscosity values were estimated by the Mamedov equation, as proposed by Cano-Gómez et al. [44].

The expected dependency of $D_{12}$ with T, P, and Stokes−Einstein abscissae $\left(T/\mu_{1}\right)$ was generally conserved, as can be observed in Table 1 and in Figure 2. $D_{12}$ increases with rising temperature due to the higher internal energy and higher free volume of the system, which facilitate diffusion. Raising the pressure decreases the free volume of the solvent and increases the energy required for the solute to escape from the force field generated by the solvent, thus penalizing its diffusion [41,42,43]. As for the Stokes–Einstein abscissae, a linear relation between $D_{12}$ and $T/\mu_{1}$ was found in both cases, with $R^{2}$ values of 0.9737 and 1.000 for quercetin and gallic acid, respectively.

### 2.2. D_12_ of Organic Solutes in Liquid Ethanol: Oxygen’s Radius Validation and Cases of Applicability

**Figure 3:**
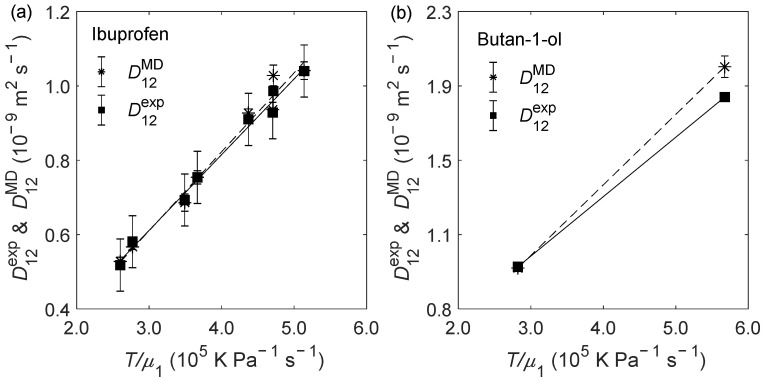
Experimental ($D_{12}^{\exp}$, ■) and computed ($D_{12}^{\mathrm{MD}}$, *) diffusion coefficients in liquid ethanol versus Stokes–Einstein abscissae ($T/\mu_{1}$) for (**a**) ibuprofen and (**b**) butan-1-ol. The viscosity values were estimated by the Mamedov equation, as proposed by Cano-Gómez et al. [44].

For validation of the new proposed parametrization, we selected two OH-bearing systems with polar ends, namely, ibuprofen, an organic acid, and butan-1-ol, a primary alcohol, both in liquid ethanol. Alternative compounds for testing would be phenol and benzoic acid, but, unfortunately, the experimental diffusivities for these two compounds are scarce. As with the previous two systems, when employing ethanol’s $\sigma_{\mathrm{OH}}$ = 0.312 nm, the $D_{12}$ values deviate up to 32.24% from the experimental value for ibuprofen and up to 33.20% for butan-1-ol at the tested conditions (see Table S2 for detailed results). When using $\sigma_{\mathrm{OH}}$ = 0.306 nm, the maximum deviation decreased to 4.26% and 8.86% for ibuprofen and butan-1-ol, respectively, with AARD = 1.55% and ARD = 0.70% for ibuprofen and AARD = 4.81% and ARD = 4.05% for butan-1-ol, confirming the improvements introduced by the proposed parameterization, as observed for the previous two systems. Furthermore, the dependency with T and P was also conserved, as can be evidenced by the results in Table 2. As for the Stokes–Einstein relation, depicted in Figure 3, once again, a linear relation was found between $D_{12}^{\mathrm{MD}}$ and $T/\mu_{1}$ with $R^{2}$ = 0.9781 (experimental $R^{2}$ = 0.9891) for ibuprofen and for butan-1-ol, for which only two points were computed. 

So far, the new parametrization improvements are independent of the actual functional group of the solute, i.e., alcohol (OH) or organic acid (COOH), confirming the results for quercetin (with five OH groups) and gallic acid (with three OH groups and one COOH group). To further study the validity of this hypothesis, additional simulations were performed for compounds without the OH moiety, namely, two hydrogen-bond-acceptor solutes (propanone and butanal) and two non-polar solutes (propane and benzene).

The $D_{12}^{\mathrm{MD}}$ values of propanone and butanal were computed in previous work [20], where the $\sigma_{\mathrm{OH}}$ value of 0.312 nm yielded satisfactory results, with AARD values between 9.48% and 12.18% and ARD between 8.37% and 12.18% for the ketones studied. For aldehydes, the situation was similar, with AARD between 6.30% and 9.11% and ARD between 1.00% and 5.67%. All the simulations were carried out at temperatures between 303.15 K and 333.15 K and pressures up to 150 bar [20]. In this work, to test the new proposed $\sigma_{\mathrm{OH}}$ value, two different temperatures (303.15 K and 333.15 K) and one value of pressure (1 bar) were simulated, for both solutes, using the computational procedure reported by Zêzere et al. [20], this time with 3 ns of equilibrium and 3 ns of production. Only one value of pressure was simulated since no strong dependence between P and RD has been found, either in Ref. [20] or in the present study. For propanone, at 303.15 K, the newly computed $D_{12}^{\mathrm{MD}}$ value shows higher absolute deviation (RD = −17.63%) than the one computed with $\sigma_{\mathrm{OH}}$ = 0.312 nm, for which RD = 1.55%. However, at 333.15 K, the situation is quite the opposite, with the newly computed $D_{12}^{\mathrm{MD}}$ value achieving a lower absolute RD value (RD = 2.08%) than the one achieved with $\sigma_{\mathrm{OH}}$ = 0.312 nm (RD = 23.53%). In this particular case, while the absolute RD value at higher T is lower, when setting $\sigma_{\mathrm{OH}}$ = 0.306 nm, it comes at a cost of degraded performance at lower T, i.e., 303.15 K. The same behavior is verified for the aldehyde (butanal), for which the value of RD increases (from −5.15% to −24.48%) at 303.15 K and 1 bar and decreases (from 16.94% to −0.07%) at 333.15 K and 1 bar. Hence, for propanone and butanal, additional corrections are needed to translate the experimental dependence of the diffusivities with temperature, which affects also the data calculated with the ethanol’s $\sigma_{\mathrm{OH}}$ value of 0.312 nm [20].

As for the non-polar solutes (benzene and propane), the $D_{12}^{\mathrm{MD}}$ values obtained with $\sigma_{\mathrm{OH}}$ = 0.306 nm present larger deviations in relation to $D_{12}^{\exp}$ than when the $\sigma_{\mathrm{OH}}$ = 0.312 nm value is used. For benzene, at 313.15 K and 1 bar, $D_{12}^{\mathrm{MD}}$ = 2.28 × 10^−9^ ± 0.08 × 10^−9^ m^2^ s^−1^ when considering $\sigma_{\mathrm{OH}}$ = 0.312 nm and $D_{12}^{\mathrm{MD}}$ = 1.85 × 10^−9^ ± 0.02 × 10^−9^ m^2^ s^−1^ when considering $\sigma_{\mathrm{OH}}$ = 0.306 nm, which correspond to RD of 0.00% and −18.86%, respectively. As for propane, the simulations at 323.15 K and 103 bar achieved similar results with $D_{12}^{\mathrm{MD}}$ = 3.10 × 10^−9^ ± 0.05 × 10^−9^ m^2^ s^−1^ with $\sigma_{\mathrm{OH}}$ = 0.312 nm and $D_{12}^{\mathrm{MD}}$ = 2.55 × 10^−9^ ± 0.08 × 10^−9^ m^2^ s^−1^ when $\sigma_{\mathrm{OH}}$ = 0.306 nm, corresponding to RD values of 3.68% and −14.72%, respectively.

To conclude this section, it is now safe to assume that the optimal $\sigma_{\mathrm{OH}}$ value is tied to the solute containing OH, or not, independently of the functional group where the atoms are. Considering the studied cases, when the solute is protic (i.e., the solute can participate in hydrogen bonds as donor and as acceptor), best results are obtained when $\sigma_{\mathrm{OH}}$ takes the value of 0.306 nm for the $D_{12}^{\;}$ calculation. Conversely, when the solute is non-polar, the value for $\sigma_{\mathrm{OH}}$ should be 0.312 nm. In the case of solutes with the ability to be hydrogen bond acceptors but not hydrogen bond donors, e.g., ketones and aldehydes, the preferred $\sigma_{\mathrm{OH}}$ should be 0.312 nm since any lower value would cause RD to increase at lower T, despite decreasing RD at higher T values.

### 2.3. $D_{11}^{\;}$ of Liquid Ethanol

**Figure 4:**
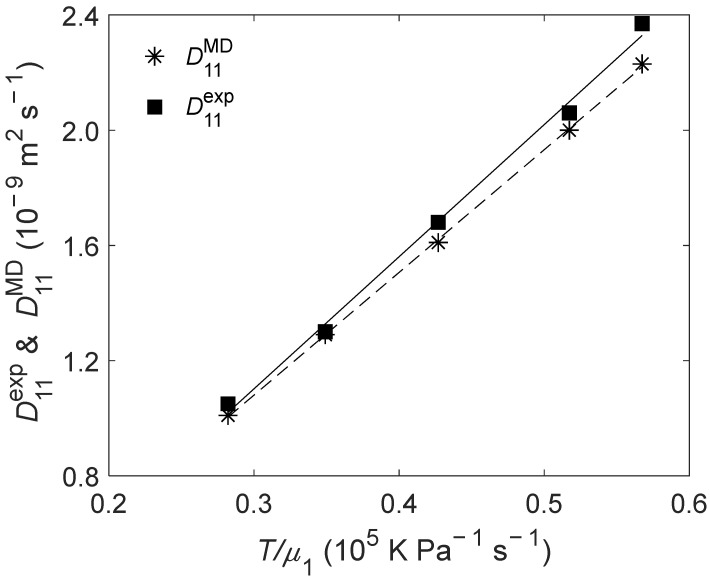
Experimental ($D_{11}^{\exp}$, ■) and computed ($D_{11}^{\mathrm{MD}}$, *) self-diffusion coefficients of ethanol versus Stokes–Einstein abscissae. The viscosity values were estimated by the Mamedov equation, as proposed by Cano-Gómez et al. [44].

The influence of the $\sigma_{\mathrm{OH}}$ parameter was further tested on the computation of $D_{11}^{\;}$ of liquid ethanol at 1 bar and at T between 298.15 K and 333.15 K. When parametrizing ethanol with $\sigma_{\mathrm{OH}}$ = 0.312 nm, the achieved RD values were between 24.05% and 35.38%, with AARD = 30.62% and ARD = 30.62% (see Table S3 for more details). However, and similarly to the results for protic solutes, when parametrizing ethanol with $\sigma_{\mathrm{OH}}$ = 0.306 nm, the achieved results drastically improve with the new-found RD between −5.91% and −0.77%, AARD = −3.51%, and ARD of the same value, as reported in Table 3. Furthermore, and similarly to what was verified for the $D_{12}^{\;}$ calculation, the computed $D_{11}^{\;}$ of ethanol follows the expected trends (i.e., increasing with T and the Stokes–Einstein coordinates). As depicted in Figure 4, there is a linear relation between $D_{11}$ and $T/\mu_{1}$ with $R^{2}$ = 0.9997 (experimental value of 0.9958).

### 2.4. Influence of the Oxygen’s Energy Parameter

The influence of the Lennard-Jones energy parameter of ethanol’s oxygen ($\varepsilon_{\mathrm{OH}}$) in the $D_{11}^{\mathrm{MD}}$ of ethanol and $D_{12}^{\mathrm{MD}}$ of quercetin and benzene in ethanol was also analyzed. It was found that increasing by 20% the value of $\varepsilon_{\mathrm{OH}}$ (i.e., the value was made equal to the one in the TraPPE-UA force field [26]) for calculations of $D_{11}^{\mathrm{MD}}$ of ethanol at 298.15 K and 1 bar, with $\sigma_{\mathrm{OH}}$ = 0.306 nm, originated a minor improvement of the diffusivity (RD changed from −3.81% to 1.90%) without negatively affecting the density (RD was barely the same), suggesting that additional improvements can be made by fine tuning the value of $\varepsilon_{\mathrm{OH}}$. In the case of the original $\sigma_{\mathrm{OH}}$ = 0.312 nm, the increase by 20% of $\varepsilon_{\mathrm{OH}}$ led to an increase of RD from 33.33% to 38.10%, while a decrease by 20% of $\varepsilon_{\mathrm{OH}}$ decreased RD from 33.33% to 25.71%, which suggests that fine tuning of the energy scale with the original radius value will hardly lead to a significant improvement in the $D_{11}^{\mathrm{MD}}$ of ethanol.

As it happened with the $D_{11}^{\mathrm{MD}}$ of ethanol, a systematic shift of the RD to more positive values was found for the protic compound quercetin when the value of $\varepsilon_{\mathrm{OH}}$ was increased by 20%. For example, when employing $\sigma_{\mathrm{OH}}$ = 0.306 nm, the RD for the calculated diffusivity, at 1 bar and 303.15 K, changed from −6.30% to 1.55%, while the value at 1 bar and 323.15 K changed from 3.13% to −7.10%. However, such systematic variation was not found in the case of the non-polar benzene molecule. In fact, when employing $\sigma_{\mathrm{OH}}$ = 0.306 nm, the RD for the calculated diffusivity, at 1 bar and 313.15 K, changed from −18.86% to −19.30%.

The results above suggest that it may be possible to slightly decrease the RD values between calculated and experimental results upon tuning the $\varepsilon_{\mathrm{OH}}$ parameter but also that two different $\sigma_{\mathrm{OH}}$ values are needed for protic and non-polar solutes, i.e., it will be difficult to find a unique solution for all kinds of solutes. Hence, a new $\sigma_{\mathrm{OH}}$ of ethanol’s oxygen atom is proposed while fixing the energy parameter ($\varepsilon_{\mathrm{OH}}$).

### 2.5. Equilibrium Properties of Ethanol

Typically, two properties used for the force-field calibration and validation are the enthalpy of vaporization ($\Delta H_{\mathrm{vap}}$) and density (ρ). Contrary to what was verified in the case of diffusivities, when the original value $\sigma_{\mathrm{OH}}$ = 0.312 nm is used for the calculation of $\Delta H_{\mathrm{vap}}$, the computed value (42.0 kJ mol^−1^) compares well with the experimental result (42.3 ± 0.4 kJ mol^−1^ [45]), with RD = −0.71%. When the reparametrized value ($\sigma_{\mathrm{OH}}$ = 0.306 nm) is used, the computed value of $\Delta H_{\mathrm{vap}}$ increases to 44.8 kJ mol^−1^, which is 5.91% higher than the one computed with $\sigma_{\mathrm{OH}}$ = 0.312 nm. This difference is mainly due to the computed potential energy of the liquid phase ($U_{\mathrm{liquid}}$) being around 13% higher when using $\sigma_{\mathrm{OH}}$ = 0.312 nm.

As for density, the values computed with $\sigma_{\mathrm{OH}}$ = 0.306 nm are always higher than the ones computed with $\sigma_{\mathrm{OH}}$ = 0.312 nm. This trend was already expected since, as stated before, a smaller $\sigma_{\mathrm{OH}}$ value means the molecules may be closer to each other; hence, density increases. As for the density values computed with $\sigma_{\mathrm{OH}}$ = 0.306 nm, RD values ranged between 1.59% and 2.58% for 298.15 K ≤ T ≤ 333.15 K at 1 bar. At a higher-pressure of 300 bar, the density values, computed at 308.15 K and 333.15 K, were also higher than the experimental ones with RD of 2.77% and 1.41%, respectively. Globally, this translates into an AARD = 2.12% and ARD of same value, which are both higher than the results obtained when using the original $\sigma_{\mathrm{OH}}$ value (AARD = 0.46% and ARD = 0.34%). These results are summarized in Table 4, and comparison between these density values and the ones obtained with $\sigma_{\mathrm{OH}}$ = 0.312 nm can be found in Table S4.

## 3. Materials and Methods

### 3.1. Database

**Figure 5:**
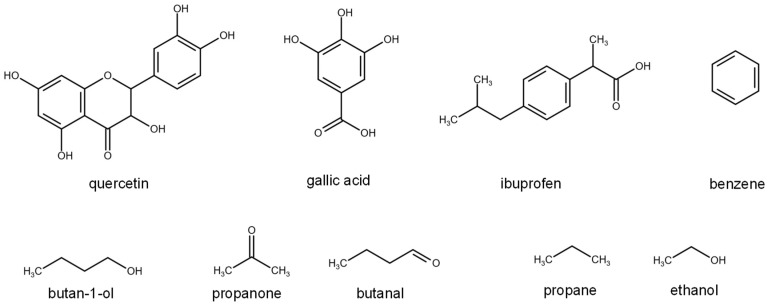
Structural formulas of the compounds studied in this work.

The database compiled for evaluation and optimization of the ethanol’s oxygen diameter ($\sigma_{\mathrm{OH}}$) is summarized in Table 5 for solute/ethanol or pure ethanol systems. Six polar-solute systems (namely, quercetin, gallic acid, ibuprofen, butan-1-ol, propanone, and butanal, with structural formulas in Figure 5) and two systems comprising non-polar solutes (benzene and propane, also shown in Figure 5) were considered. The first two polar-solutes (i.e., quercetin and gallic acid) were used for optimization of the $\sigma_{\mathrm{OH}}$ value, while the second pair of polar solutes (i.e., ibuprofen and butan-1-ol) was selected for validation of the new proposed value. The remaining polar (i.e., propanone and butanal) alongside the non-polar solutes were chosen to investigate the initial hypothesis that OPLS-AA overestimates $D_{12}$ of protic solutes. Ethanol properties, such as $D_{11}$, density (ρ), and enthalpy of vaporization ($\Delta H_{\mathrm{vap}}$) were also included. 

All the results calculated in this work were evaluated in terms of relative deviations (RD), average relative deviations (ARD), and average absolute relative deviations (AARD) calculated by:

(1) $$\mathrm{RD}\left(\%\right)=100\frac{X^{\mathrm{MD}}-X_{\;}^{\exp}}{X_{\;}^{\exp}}$$

(2) $$\mathrm{ARD}\left(\%\right)=\frac{100}{NDP}\overset{NDP}{\underset{i=1}{\sum }}{\left(\frac{X^{\mathrm{MD}}-X_{\;}^{\exp}}{X_{\;}^{\exp}}\right)}_{i}{}_{\;}$$

(3) $$\mathrm{AARD}\left(\%\right)=\frac{100}{NDP}\overset{NDP}{\underset{i=1}{\sum }}{\left|\frac{X^{\mathrm{MD}}-X_{\;}^{\exp}}{X_{\;}^{\exp}}\right|}_{i}$$

in which X is the property under study, NDP is the number of data points, and the superscripts MD and exp represent the computed and experimental property, respectively.

### 3.2. Molecular Dynamics Simulation Procedure

The classical MD simulations were carried out with the GROMACS 2019 code [63,64,65], using cubic boxes with 3500 molecules of ethanol and 4–20 molecules of the solute, corresponding to a mass fraction in the range between 0.5% and 1%. The precise number of solute molecules used for each system can be found in Supplementary Materials (Table S5).

The potential parameters used in this work for the different compounds are supplied in a separate zip file in a format (.itp) that is compatible with the GROMACS code. The equations used for calculation of the interactions and the topologies of the solute molecules can be found in Supplementary Materials (Equations (S1)–(S8)). For each temperature/pressure condition, the simulations were carried out using a published procedure by Zêzere et al. [20], with longer simulation times, based on the computational recipe proposed by Barrera and Jorge [23]. Each simulation was initialized by a steepest-descent minimization run, followed by a 100 ps simulation using the canonical ensemble (NVT) with initial velocities generated according to the Maxwell–Boltzmann distribution; next, a 100 ps run in the isothermal–isobaric ensemble (NPT) was carried out using the Berendsen coupling scheme [66]; finally, the simulation continued in the NPT ensemble up to a few nanoseconds (see below) with a time step of 0.001 ps. The last phase of the simulation was carried out using the leap-frog algorithm [67], with the box temperature and pressure being kept constant by using the V-rescale thermostat [68] and the Parrinello–Rahman barostat [69], respectively. The choice for the NPT ensemble was to certify that the simulations were performed at the same pressure and temperature conditions used to measure experimental data. Nevertheless, in previous work, it was found that diffusivities calculated from NVT and NPT simulations are very similar, with the main difference being the average pressure of the simulation [20]. Additionally, the LINCS algorithm was used to constrain the bond lengths, and a cut-off distance of 1.4 nm was adopted, as tested in a previous work [20], for both van der Walls and Coulomb interactions. The Particle–Mesh Ewald (PME) [70] summation was selected for the long-range electrostatic interactions. The simulation was carried out using the standard periodic boundary conditions, applying long-range dispersion corrections for energy and pressure. The compressibility values were taken from the literature [71] or estimated using published correlations [59]. As for the duration of the simulations, these were adjusted according to the desired property, as indicated in Section 3.3 and Section 3.4.

### 3.3. Self-Diffusion and Binary Diffusion Coefficients

The diffusion coefficients ($D_{11}$ or $D_{12}$) were calculated by the Einstein relation of the mean square displacement (MSD) of the random motion of a molecule [72]:

(4) $$D_{11}\;\mathrm{or}\;D_{12}=\underset{t\to \infty }{\lim}\frac{\langle {\left[r\left(t_{0}+t\right)-r\left(t_{0}\right)\right]}^{2}\rangle }{6t}$$

in which $t_{0}$ is the time origin, t is the time elapsed from $t_{0}$, and *r* is the molecule/atom position. The average, represented by the angled brackets, was calculated using all molecules in the simulation and all time origins. Once the MSD as a function of time was known, a simple linear least-squares regression was performed between 50 and 100 ps, as shown previously, to yield accurate results [20].

For the $D_{12}$ calculations, the final NPT ensemble was carried out during 40 ns of which the first 20 ns were discarded to assure proper equilibration of the dynamics [20]. The final $D_{12}$ value was obtained from averaging the $D_{12}$ results of three independent simulation replicas.

As for $D_{11}$ calculations, the simulations were carried out with the setup previously described, with 10 ns of equilibration and 15 ns of production, and only one simulation was considered for the final $D_{11}$ value. Typically, the computed $D_{11}$ values are affected by the finite size of the simulation box. Hence, a correction should be introduced to overcome this limitation which, according to Yeh and Hummer [73], can be conducted by performing a linear regression between $D_{11}^{\mathrm{MD},L}$, the self-diffusion coefficient computed from a simulation box with finite length L, and 1/L. Accordingly:

(5) $$D_{11}^{\mathrm{MD},L}=m\times \frac{1}{L}+D_{11}^{\mathrm{MD},\infty }$$

where $D_{11}^{\mathrm{MD},\infty }$ is the self-diffusion coefficient computed from the simulation box with infinite length (*y*-intercept at 1/*L* = 0), and m is the slope. Alternatively, a hydrodynamic correction can be directly applied [73]:

(6) $$D^{\mathrm{YH}}\left(T,\mu_{1},L\right)=\frac{\xi k_{B}T}{6\pi \mu_{1}L}$$

(7) $$D_{11}^{\mathrm{MD},\infty }=D_{11}^{\mathrm{MD},L}+D_{\;}^{\mathrm{YH}}\left(T,\mu_{1},L\right)$$

in which $D^{\mathrm{YH}}\left(T,\mu_{1},L\right)$ is the Yeh and Hummer hydrodynamic correction of $D_{11}^{\mathrm{MD},L}$, $k_{B}$ is the Boltzmann constant (1.380649 × 10^−23^ m^2^ kg s^−2^ K^−1^), ξ is a dimensional constant of value 2.837297, and $\mu_{1}$ the viscosity. This second approach was adopted in this work since it is computationally cheaper, and the $D_{11}^{\mathrm{MD},\infty }$ results are equivalent to those obtained by the linear regression method, as depicted in Figure S1 (see Supplementary Materials). A total of 1500 ethanol molecules were used per simulation at each condition. 

### 3.4. Calculation of Equilibrium Properties

#### 3.4.1. Enthalpy of Vaporization

The enthalpy of vaporization was computed by:

(8) $$\Delta H_{\mathrm{vap}}=U_{\mathrm{gas}}-U_{\mathrm{liquid}}+R_{g}T$$

in which $U_{\mathrm{gas}}$ is the potential energy of the vapor phase, $U_{\mathrm{liquid}}$ the potential energy of the liquid phase, and $R_{g}$ the ideal gas constant ($R_{g}$ = 8.314 J K^−1^ mol^−1^). To calculate $U_{\mathrm{liquid}}$, we used the last 15 ns of the simulation used to compute $D_{11}^{\;}$. The Barrera and Jorge [23] procedure was used to calculate $U_{\mathrm{gas}}$, with one molecule being placed inside a cubic box (15 nm × 15 nm × 15 nm), with no boundary conditions and all cutoff radii set to 0. The simulation was carried out in NVT using the leap-frog stochastic dynamics integrator, which adds a friction and a noise term to Newton’s equation of motion [74]. The run was carried out for 50 ns, and the first 10 ns of the simulation were discarded.

#### 3.4.2. Density

The densities of pure ethanol systems were calculated from the last 15 ns of the trajectories. For conditions for which no $D_{11}^{\;}$ was computed, the simulations were carried out with the setup used for $D_{11}^{\;}$ calculation.

## 4. Conclusions

The OPLS-AA force field was used in the calculation of tracer diffusion coefficients ($D_{12}$) of solutes in ethanol and of self-diffusion coefficients ($D_{11}$) of ethanol from molecular dynamics simulations carried out at different temperature and pressure conditions. It was found that when the oxygen atom of ethanol considers the original OPLS-AA diameter, it yields high deviations of $D_{11}$ of ethanol and of $D_{12}$ of protic solutes in ethanol.

In order to correct such deviations, the diameter of the oxygen atom ($\sigma_{\mathrm{OH}}$) of ethanol was reoptimized by targeting $D_{12}$ of quercetin and of gallic acid (both protic solutes) in liquid ethanol, at 303.15 K ≤ T ≤ 323.15 K and P up to 150 bar, and 303.15 K ≤ T ≤ 333.15 K and P = 1 bar, respectively. With the new optimized value, i.e., $\sigma_{\mathrm{OH}}$ = 0.306 nm, the comparison with the experiment was substantially improved, with an average absolute relative deviation (AARD) of 3.71% and average relative deviation (ARD) of −0.04% for quercetin and AARD = 4.59% and ARD = 1.05% for gallic acid.

Significant improvements were also found for the computed $D_{12}$ of ibuprofen in liquid ethanol at 298.15 K ≤ T ≤ 333.15 K and P up to 300 bar, with AARD = 1.55% and ARD = 0.70%, and for butan-1-ol at 298.15 K ≤ T ≤ 333.15 K and P = 1 bar, with AARD = 4.81% and ARD = 4.05%. A mixed behavior was observed for propanone and butanal for which the results improved at T = 333.15 K but significantly deteriorated at T = 303.15 K. However, in the case of non-polar solutes such as propane or benzene, the performance worsened with the new value at all the tested conditions. Regarding the $D_{11}$ of ethanol, the results vastly improved, having achieved AARD and ARD values of 3.51% and −3.51%, respectively, at the tested conditions.

In all the test systems, when computing either $D_{12}$ or $D_{11}$, the expected trends with T, P, and Stokes–Einstein abscissae were always conserved.

Finally, the influence of the new value was evaluated in both enthalpy of vaporization ($\Delta H_{\mathrm{vap}}$) and density of ethanol, achieving slightly worse results in both cases with RD = 5.91% for $\Delta H_{\mathrm{vap}}$ and AARD = 2.12% and ARD of same value for density. Hence, the value of $\sigma_{\mathrm{OH}}$ = 0.306 nm is recommended only for the calculations of $D_{12}$ of protic solutes containing OH in pure liquid ethanol and $D_{11}$ estimation.

## Figures and Tables

**Table 1:** Experimental ($D_{12}^{\exp}$) and computed ($D_{12}^{\mathrm{MD}}$) diffusion coefficients of quercetin and gallic acid in liquid ethanol at various temperatures and pressures. The computer simulations used $\sigma_{\mathrm{OH}}$ = 0.306 nm for ethanol, and the calculated diffusivities were evaluated in comparison with experimental data in terms of the relative deviation (RD), average relative deviation (ARD), and average absolute relative deviation (AARD).

Solute	T (K)	P (bar)	$D_{12}^{exp}\pm \Delta D_{12}^{exp}$ (10^−9^ m^2^ s^−1^)	$D_{12}^{MD}\;\pm \Delta D_{12}^{MD}$ (10^−9^ m^2^ s^−1^)	RD (%)
Quercetin	303.15	1	0.459 ± 0.003	0.430 ± 0.014	−6.30
	303.15	150	0.414 ± 0.002	0.409 ± 0.010	−1.21
	323.15	1	0.681 ± 0.002	0.702 ± 0.003	3.13
	323.15	150	0.616 ± 0.003	0.642 ± 0.036	4.22
	ARD = −0.04%				
	AARD = 3.71%				
Gallic acid	303.15	1	0.508 ± 0.009	0.481 ± 0.014	−5.31
	323.15	1	0.758 ± 0.006	0.776 ± 0.028	2.37
	333.15	1	0.905 ± 0.011	0.960 ± 0.028	6.08
	ARD = 1.05%				
	AARD = 4.59%				

**Table 2:** Experimental ($D_{12}^{\exp}$) and computed ($D_{12}^{\mathrm{MD}}$) diffusion coefficients of ibuprofen and butan-1-ol in liquid ethanol at various temperatures and pressures. The computer simulations used $\sigma_{\mathrm{OH}}$ = 0.306 nm for ethanol, and the calculated diffusivities were evaluated in comparison with experimental data in terms of the relative deviation (RD), average relative deviation (ARD), and average absolute relative deviation (AARD).

Solute	T (K)	P (bar)	$D_{12}^{exp}\pm \Delta D_{12}^{exp}$ (10^−9^ m^2^ s^−1^)	$D_{12}^{MD}\;\pm \Delta D_{12}^{MD}$ (10^−9^ m^2^ s^−1^)	RD (%)
ibuprofen	298.15	100	0.518 ± 0.070	0.528 ± 0.011	1.93
	308.15	1	0.693 ± 0.070	0.686 ± 0.023	−1.01
	308.15	300	0.581 ± 0.070	0.567 ± 0.007	−2.41
	323.15	1	0.928 ± 0.070	0.936 ± 0.020	0.86
	323.15	300	0.754 ± 0.070	0.754 ± 0.018	0.00
	333.15	100	1.04 ± 0.07	1.04 ± 0.02	0.10
	333.15	200	0.986 ± 0.070	1.03 ± 0.03	4.26
	333.15	300	0.910 ± 0.070	0.927 ± 0.011	1.87
ARD = 0.70%					
AARD = 1.55%					
butan-1-ol	298.15	1	0.927	0.920 ± 0.011	−0.76
	333.15	1	1.84	2.00 ± 0.06	8.86
ARD = 4.05%					
AARD = 4.81%					

**Table 3:** Experimental ($D_{11}^{\exp}$) and computed ($D_{11}^{\mathrm{MD}}$) self-diffusion coefficient of ethanol. The computer simulations used $\sigma_{\mathrm{OH}}$ = 0.306 nm for ethanol, and the calculated diffusivities were evaluated in comparison with experimental data in terms of the relative deviation (RD), average relative deviation (ARD), and average absolute relative deviation (AARD).

T (K)	P (bar)	$D_{11}^{exp}$ (10^−9^ m^2^ s^−1^)	$D_{11}^{MD}$ (10^−9^ m^2^ s^−1^)	RD (%)
298.15	1	1.05	1.01	−3.81
308.15	1	1.30	1.29	−0.77
318.15	1	1.68	1.61	−4.17
328.15	1	2.06	2.00	−2.91
333.15	1	2.37	2.23	−5.91
ARD = −3.51%				
AARD = 3.51%				

**Table 4:** Ethanol’s experimental $\left(\rho_{\;}^{\exp}\right)$ and computed ($\rho^{\mathrm{MD}}$) density and respective relative deviation (RD). The computer simulations used $\sigma_{\mathrm{OH}}$ = 0.306 nm for ethanol.

T (K)	P (bar)	$\rho_{\;}^{exp}$ (kg m^−3^)	$\rho^{MD}$ (kg m^−3^)	RD (%)
298.15	1	786	806	2.54
308.15	1	776	796	2.58
308.15	300	795	817	2.77
318.15	1	767	785	2.08
328.15	1	759	773	1.84
333.15	1	754	768	1.59
333.15	300	782	793	1.41
ARD = 2.12%				
AARD = 2.12%				

**Table 5:** Experimental properties studied for binary (ethanol/solute) or unary (ethanol) systems, number of data points (NDP), temperature (T) range, pressure (P) range, and data sources.

System	Property	NDP	T (K)	P (bar)	Source
EtOH/quercetin	$D_{12}$	4	303.15–323.15	1–150	[46]
EtOH/gallic acid	$D_{12}$	3	303.15–333.15	1	[5]
EtOH/ibuprofen	$D_{12}$	8	298.15–333.15	1–300	[4]
EtOH/butan-1-ol	$D_{12}$	2	298.15–333.15	1	[47]
EtOH/propanone	$D_{12}$	2	298.15–333.15	1	[48]
EtOH/butanal	$D_{12}$	2	298.15–333.15	1	[48]
EtOH/benzene	$D_{12}$	1	313.15	1	[47,49]
EtOH/propane	$D_{12}$	1	323.15	103	[50]
EtOH	$D_{11}$	5	298.15–333.15	1	[51,52,53,54,55,56,57,58]
EtOH	ρ	8	298.15–333.15	1–300	[59,60,61,62]
EtOH	$\Delta H_{\mathrm{vap}}$	1	298.15	1	[45]

## Data Availability

The raw/processed data required to reproduce the above findings cannot be shared at this time due to technical/time limitations.

## Supplementary Materials

The supporting information can be downloaded at: https://www.mdpi.com/article/10.3390/ijms24087316/s1.
