# Supplementary material for: Influence of Ethanol Parametrization on Diffusion Coefficients Using OPLS-AA Force Field
Source: Int J Mol Sci. 2023 Apr 15;24(8):7316. doi: 10.3390/ijms24087316 (PMC10138630; doi:10.3390/ijms24087316)
Supplement: Supplementary file 1 [file ijms-24-07316-s001.zip › SM_2023_03_31.pdf]

Supplementary Materials of

Influence of ethanol parametrization on diffusion coefficients using OPLS-AA force field

Bruno Zêzere[a], Tiago V. B. Fonseca[a], Mário M. Q. Simões[b], Inês Portugal[a], Carlos M. Silva[a]*, José R. B. Gomes[a]*

[a]CICECO – Aveiro Institute of Materials, Department of Chemistry, University of Aveiro, Campus Universitário de Santiago, 3810-193 Aveiro, Portugal

[b]LAQV-REQUIMTE, Department of Chemistry, University of Aveiro, Campus Universitário de Santiago, 3810-193 Aveiro, Portugal

* Corresponding authors: carlos.manuel@ua.pt; jrgomes@ua.pt

Potential energy functions

The terms whose sum gives in OPLS-AA the potential energy are of two types, binding and non-binding.

Binding terms correspond to 1) covalent bonds ($V_{\mathrm{stretching}}$):

$$V_{\mathrm{stretching}} = k_{\mathrm{ij}}/2\left(r_{\mathrm{ij}} - r_0\right)^2 \tag{S1}$$

in which r_{ij} is the distance between the particle i and j, r_0 is the equilibrium bond length and k_{ij} is the stretching force constant; 2) angles between two covalent bonds of the same atom (V_{angle}):

$$V_{\mathrm{angle}} = k_{\mathrm{ikj}}/2\left(\theta_{\mathrm{ikj}} - \theta_0\right)^2 \tag{S2}$$

in which θ_{ikj} is the angle between the particles i, k and j, θ_0 is the equilibrium angle and k_{ikj} is the bending force constant; or 3) angles between two planes, each of two covalent bonds of the same atom, with a common covalent bond (dihedral angles), which can be proper ($V_{\mathrm{proper\ dihedrals}}$):

$$V_{\mathrm{proper\ dihedrals}} = \sum_{\mathrm{n}=0}^{5} k_{\mathrm{n}}\left(\cos\left(\theta_{\mathrm{ijkl}} - 180\right)\right)^{\mathrm{n}} \tag{S3}$$

in which θ_{ijkl} is the torsion angle between i, j, k and l, and k_{n} are the function coefficients, or improper ($V_{\mathrm{improper\ dihedrals}}$):

$$V_{\mathrm{improper\ dihedrals}} = k_{\mathrm{ijkl}}\left(1 + \cos\left(2\,\theta_{\mathrm{ijkl}} - \theta_0\right)\right)^{\mathrm{n}} \tag{S4}$$

in which k_{ijkl} is the force constant from the internal torsions.

Non-binding terms ($V_{\mathrm{non-bonded}}$) correspond to pairs of atoms covalently unbound to each other or the same atom and were calculated accounting for the sum between the van der Waals ($V_{\mathrm{van\ der\ Waals}}$) and Coulombic ($V_{\mathrm{Coulomb}}$) interactions. The van der Waals are calculated as:

$$V_{\mathrm{non-bonded}} = 4\varepsilon_{\mathrm{ij}}\left[\left(\frac{\sigma_{\mathrm{ij}}}{r_{\mathrm{ij}}}\right)^{12} - \left(\frac{\sigma_{\mathrm{ij}}}{r_{\mathrm{ij}}}\right)^{6}\right] + \frac{q_{\mathrm{i}}q_{\mathrm{j}}}{4\varepsilon_{\mathrm{r}}r_{\mathrm{ij}}} \tag{S5}$$

in which $\varepsilon_{\mathrm{ij}}$ is the well depth potential energy between the particles i and j at $r_{\mathrm{ij}} = 2^{1/6}\sigma_{\mathrm{ij}}$, σ_{ij} is the distance between i and j at which the potential is null, q_{ij} (1 e = $1.602176565\times10^{-19}$ C) is the charge of particles i or j, and ε_{r} is the relative permittivity. The Coulombic are calculated as:

$$V_{\mathrm{non-bonded}} = \frac{q_{\mathrm{i}}q_{\mathrm{j}}}{4\varepsilon_{\mathrm{r}}r_{\mathrm{ij}}} \tag{S6}$$

The σ_{ij} and $\varepsilon_{\mathrm{ij}}$ are calculated from the i and j atoms σ and ε by the geometric combination rules:

$$\sigma_{\mathrm{ij}} = \left(\sigma_{\mathrm{i}}\sigma_{\mathrm{j}}\right)^{1/2} \tag{S7}$$

$$\varepsilon_{\mathrm{ij}} = \left(\varepsilon_{\mathrm{i}}\varepsilon_{\mathrm{j}}\right)^{1/2} \tag{S8}$$

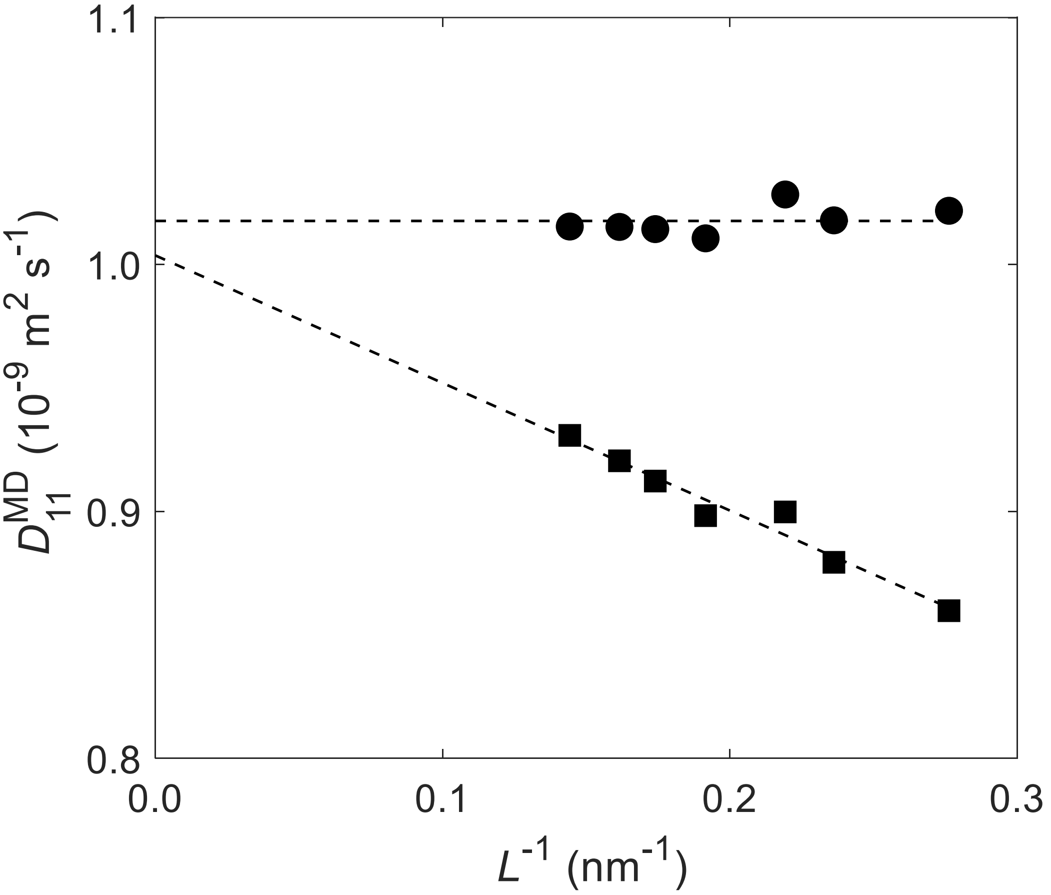

Figure S1 – Ethanol's self-diffusion coefficient *versus* $1/L$, at 298.15 K and 1 bar. The squares represent D_{11}^{MD} computed with different box length values and the circles the D_{11}^{MD} corrected with the Yeh and Hummer hydrodynamic expressions (Equations (6) and (7)).

Table S1 – Computed diffusion coefficients (D_{12}^{MD}) and relative deviations (RD) of quercetin and gallic acid at in liquid ethanol at various T, P and σ_{OH}. Uncertainties ($\Delta D_{12}^{\mathrm{MD}}$) only reported whenever 3 data points (NDP) were computed.

Solute	T (K)	P (bar)	σ_{OH} (nm)	$D_{12}^{\mathrm{MD}} \pm \Delta D_{12}^{\mathrm{MD}}$ (10^{-9} m^2 s^{-1})	NDP	RD (%)
Quercetin	303.15	1	0.312	0.581 ± 0.011	3	26.61
	323.15	1	0.312	0.897 ± 0.016	3	31.78
	303.15	1	0.310	0.529	1	15.28
	323.15	1	0.310	0.756	1	11.06
	303.15	1	0.308	0.514	1	12.01
	323.15	1	0.308	0.836	1	22.81
	303.15	1	0.307	0.469 ± 0.026	3	2.20
	303.15	150	0.307	0.421 ± 0.006	3	1.69
	323.15	1	0.307	0.725 ± 0.013	3	6.51
	323.15	150	0.307	0.670 ± 0.028	3	8.77
	303.15	1	0.306	0.430 ± 0.014	3	-6.30
	303.15	150	0.306	0.409 ± 0.010	3	-1.21
	323.15	1	0.306	0.702 ± 0.003	3	3.13
	323.15	150	0.306	0.642 ± 0.036	3	4.22
	303.15	1	0.305	0.436 ± 0.009	3	-4.99
	303.15	150	0.305	0.381 ± 0.016	3	-7.97
	323.15	1	0.305	0.667 ± 0.018	3	-2.01
	323.15	150	0.305	0.591 ± 0.012	3	-4.06
	303.15	1	0.304	0.387	1	-15.67
	323.15	1	0.304	0.615	1	-9.65
Gallic acid	303.15	1	0.312	0.656 ± 0.002	3	29.13
	323.15	1	0.312	1.02 ± 0.03	3	34.43
	333.15	1	0.312	1.22 ± 0.038	3	35.25
	303.15	1	0.310	0.595	1	17.13
	333.15	1	0.310	1.10	1	21.55
	303.15	1	0.308	0.538	1	5.91
	333.15	1	0.308	1.03	1	16.97
	303.15	1	0.307	0.491 ± 0.010	3	3.35
	323.15	1	0.307	0.809 ± 0.023	3	6.73
	333.15	1	0.307	0.978 ± 0.043	3	8.07
	303.15	1	0.306	0.481 ± 0.014	3	5.31
	323.15	1	0.306	0.776 ± 0.028	3	2.37
	333.15	1	0.306	0.960 ± 0.028	3	6.08
	303.15	1	0.305	0.458 ± 0.009	3	9.84
	323.15	1	0.305	0.722 ± 0.005	3	4.75
	333.15	1	0.305	0.916 ± 0.012	3	1.22
	303.15	1	0.304	0.442	1	12.99
	333.15	1	0.304	0.848	1	6.30

Table S2 – Computed diffusion coefficients (D_{12}^{MD}), experimental (D_{12}^{exp}) and relative deviations (RD) of the various solutes in liquid ethanol at various temperatures (T) and pressures (P), considering σ_{OH} = 0.312 nm and σ_{OH} = 0.306 nm; experimental uncertainties ($\Delta D_{12}^{\mathrm{exp}}$) only reported whenever available.

Solute	T (K)	P (bar)	$D_{12}^{\mathrm{exp}} \pm \Delta D_{12}^{\mathrm{exp}}$ (10^{-9} m^2 s^{-1})	σ_{OH} = 0.312 nm<br>$D_{12}^{\mathrm{MD}} \pm \Delta D_{12}^{\mathrm{MD}}$ (10^{-9} m^2 s^{-1})	RD (%)	σ_{OH} = 0.306 nm<br>$D_{12}^{\mathrm{MD}} \pm \Delta D_{12}^{\mathrm{MD}}$ (10^{-9} m^2 s^{-1})	RD (%)
ibuprofen	298.15	100	0.518 ± 0.070	0.685 ± 0.16	32.24	0.528 ± 0.011	1.93
	308.15	1	0.693 ± 0.070	0.877 ± 0.21	26.55	0.686 ± 0.023	-1.01
	308.15	300	0.581 ± 0.070	--	--	0.567 ± 0.007	-2.41
	323.15	1	0.928 ± 0.070	1.18 ± 0.2	26.94	0.936 ± 0.020	0.86
	323.15	300	0.754 ± 0.070	--	--	0.754 ± 0.108	0.00
	333.15	100	1.04 ± 0.07	1.32 ± 0.44	27.12	1.04 ± 0.02	0.10
	333.15	200	0.986 ± 0.070	--	--	1.03 ± 0.03	4.26
	333.15	300	0.910 ± 0.070	--	--	0.927 ± 0.011	1.87
butan-1-ol	298.2	1	0.927	1.19 ± 0.01	28.48	0.920 ± 0.011	-0.76
	333.2	1	1.84	2.45 ± 0.02	33.20	2.00 ± 0.06	8.86
propanone	303.15	1	1.94 ± 0.03	1.97 ± 0.06*	1.55	1.60 ± 0.01	-17.63
	333.15	1	2.89 ± 0.06	3.57 ± 0.10*	23.53	2.95 ± 0.16	2.08
butanal	303.15	1	1.94 ± 0.07	1.84 ± 0.08*	−5.15	1.47 ± 0.03	-24.48
	333.15	1	2.68 ± 0.06	3.13 ± 0.29*	16.79	2.68 ± 0.13	-0.07
benzene	313.15	1	2.28	2.28 ± 0.08	0.00	1.85 ± 0.02	-18.86
propane	323.15	103	2.99	3.10 ± 0.05	3.68	2.55 ± 0.08	-14.72

* Data taken from [1].

Table S3 – Computed self-diffusion coefficients of ethanol (D_{11}^{MD}), experimental (D_{11}^{exp}) and relative deviations (RD) at various temperatures (T) and pressure value of 1 bar, considering σ_{OH} = 0.312 nm and σ_{OH} = 0.306 nm

T (K)	D_{11}^{exp} (10^{-9} m^2 s^{-1})	σ_{OH} = 0.312 nm<br>D_{11}^{MD} (10^{-9} m^2 s^{-1})	RD (%)	σ_{OH} = 0.306 nm<br>D_{11}^{MD} (10^{-9} m^2 s^{-1})	RD (%)
298.15	1.05	1.40	33.33	1.01	−3.81
308.15	1.30	1.76	35.38	1.29	−0.77
318.15	1.68	2.18	29.76	1.61	−4.17
328.15	2.06	2.69	30.58	2.00	−2.91
333.15	2.37	2.94	24.05	2.23	−5.91

Table S4 – Computed density of ethanol (ρ^{MD}), experimental (ρ^{exp}) and relative deviations (RD) at various temperatures (T) and pressures (P), considering σ_{OH} = 0.312 nm and σ_{OH} = 0.306 nm

T (K)	P (bar)	ρ^{exp} (kg m^{-3})	σ_{OH} = 0.312 nm<br>ρ^{MD} (kg m^{-3})	RD (%)	σ_{OH} = 0.306 nm<br>ρ^{MD} (kg m^{-3})	RD (%)
298.15	1	786	794	1.02	806	2.54
308.15	1	776	783	0.90	796	2.58
308.15	300	795	805	0.63	817	2.77
318.15	1	767	771	0.26	785	2.08
328.15	1	759	759	0.00	773	1.84
333.15	1	754	754	−0.29	768	1.59
333.15	300	782	780	−0.13	793	1.41

Table S5 – Number of solute molecules used for each system simulated.

Solute	No. of molecules
quercetin	4
gallic acid	7
ibuprofen	7
butan-1-ol	12
propanone	15
butanal	13
benzene	12
propane	20

References

[1] B. Zêzere, I. Portugal, C.M. Silva, J.R.B. Gomes, Diffusivities of ketones and aldehydes in liquid ethanol by molecular dynamics simulations, J. Mol. Liq. 371 (2023) 121068. https://doi.org/10.1016/j.molliq.2022.121068.
